# Synthetic Mesh Reconstruction of Chronic, Native Quadriceps Tendon Disruptions following Failed Primary Repair

**DOI:** 10.1155/2021/5525319

**Published:** 2021-09-15

**Authors:** Braden E. Hartline, Jacob M. Wilson, Andrew M. Schwartz, James R. Roberson, George N. Guild

**Affiliations:** ^1^Emory University Orthopaedics & Spine Hospital, 1455 Montreal Rd. E., Tucker, GA 30084, USA; ^2^University of Texas Health Science Center at Houston, 6400 Fannin St., Houston, TX 77030, USA; ^3^Emory University School of Medicine, 201 Dowman Dr., Atlanta, GA 30322, USA

## Abstract

**Case:**

Two patients presented with chronic knee extensor mechanism disruption after failed primary repairs. Both patients had minimal ambulatory knee function prior to surgical intervention and were treated with a synthetic mesh reconstruction of their extensor mechanism. Our technique has been modified from previously described techniques used in revision knee arthroplasty. At the one-year follow-up, both patients had improvement in their active range of motion and had returned to their previous activity.

**Conclusion:**

Synthetic mesh reconstruction of chronic extensor mechanism disruption is a viable technique that can be utilized as salvage for the persistently dysfunctional native knee.

## 1. Introduction

Quadriceps tendon (*QT*) ruptures occur most frequently in middle-aged males [1] and typically can be successfully treated with primary surgical repair [2, 3]. While worse surgical outcomes are associated with delays to primary repair, the overall rate of repair failure or rerupture of acute injuries remains low (approximately 2%) [2, 4, 5]. In the acute setting, *QT* injuries are typically repaired with direct tissue apposition, transosseous tunnels, or suture anchors, depending on whether the injury occurs midsubstance or at the osseotendinous interface [2, 4, 5]. Treatment of chronic ruptures or reruptures of prior repairs represents a greater surgical challenge, with no clear gold standard for reconstruction. Described options for surgical reinforcement include the use of allograft [6] and autograft tissue [7–9].

We present two cases of chronic, reruptured *QT* injuries in native knees treated with synthetic mesh reconstruction. *QT* reconstruction using this technique, typically reserved for post total knee arthroplasty (TKA) knees, resulted in favorable outcomes in both patients at the final follow-up.

## 2. Statement of Informed Consent

Both patients signed informed consent permitting us to report on their deidentified cases.

### 2.1. Case Presentation and Surgical Technique

#### 2.1.1. Case 1

**Figure 1:**
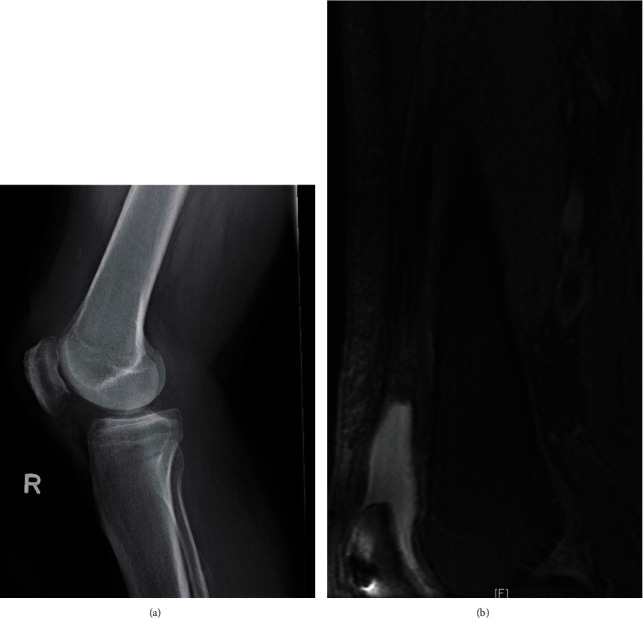
Patient 1 (a) lateral X-ray showing no significant bony injury and (b) sagittal MRI images of the distal femur showing significant *QT* disruption, tendon retraction, and postsurgical patellar changes.

An 82-year-old male with baseline function of daily jogging and a past medical history of chronic kidney disease presented with right knee pain and dysfunction. He had failed two attempts at primary quad tendon repair, first with suture anchors 4 months prior to presentation and subsequently with transosseous tunnels one month later. At presentation, he had a palpable defect just proximal to the superior patellar pole and was unable to actively straight leg raise (MRI and X-ray shown in Figure 1).

His passive ROM was 0-120, and active ROM was 70-120° (i.e., 70° extensor lag). *His Knee Society Score (KSS) was 35*. The patient was able to ambulate with his knee locked in extension with a compensatory circumduction gait.

At the 12-month follow-up after mesh reconstruction (described below), he was ambulating without assistive devices and had an active knee range of motion (ROM) of 5-120°. He had resumed light running activities and *achieved a KSS of 73*.

#### 2.1.2. Case 2

**Figure 2:**
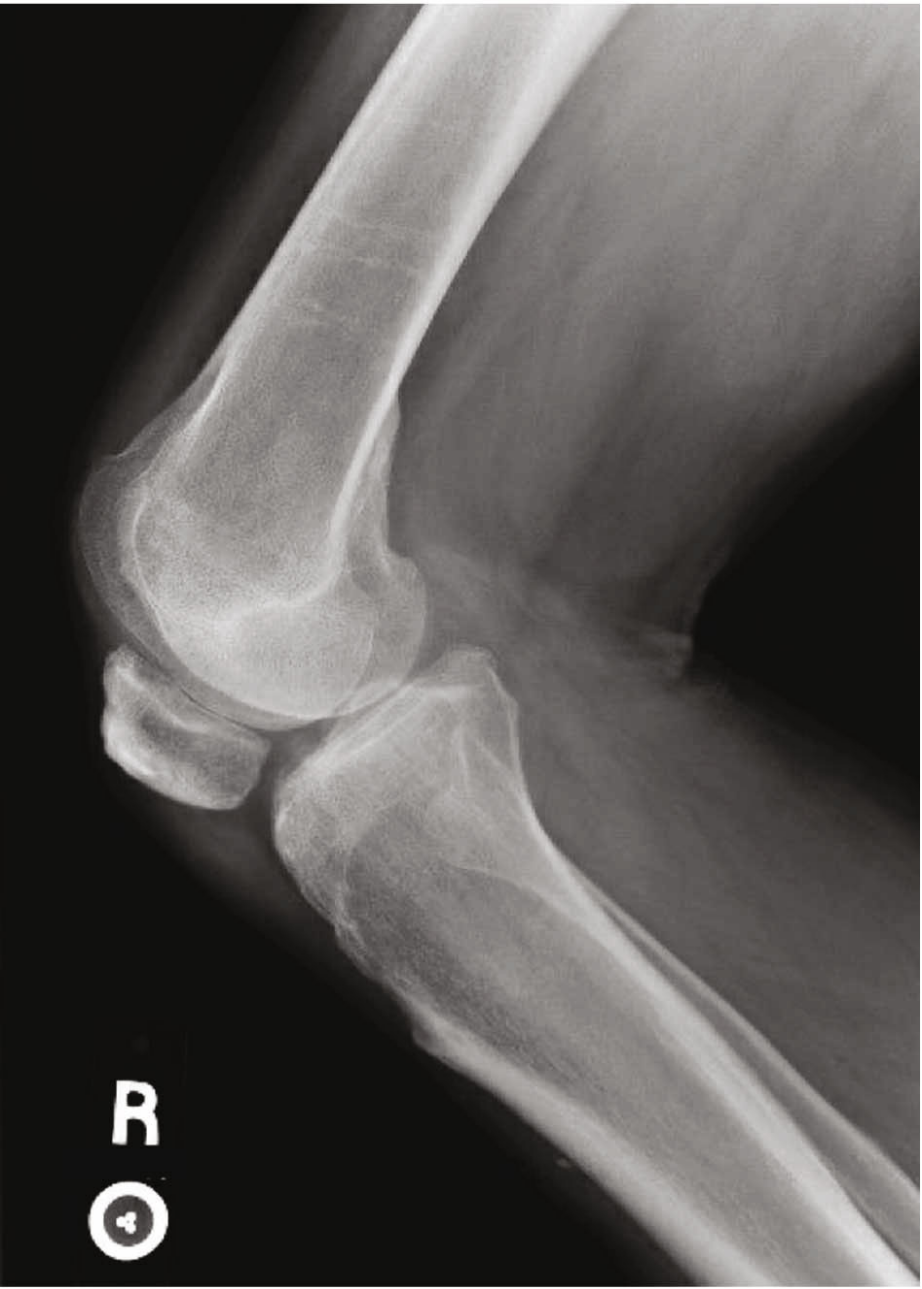
Patient 2 lateral X-ray showing significant radiographic patellar baja but no significant bony injury.

A 58-year-old male with a remote history significant for a left, traumatic above-knee amputation presented with right knee pain and dysfunction 1 year following primary *QT* repair complicated by a fall and rerupture postoperatively. He previously ambulated unassisted with a prosthesis. The patient was wheelchair-bound and had passive ROM of 0-120° and active ROM between 75 and 120°. *His KSS was 31*. His preoperative X-ray was significant for patellar baja and no fracture (Figure 2).

At the 12-month follow-up after extensor reconstruction (see below), he had returned to unassisted ambulation with his prosthesis. His passive ROM was preserved, and his active ROM had improved to 10-120 degrees and *achieved a KSS of 71*.

#### 2.1.3. Surgical Technique

The patient is positioned supine on a regular surgical bed, and a tourniquet is used. A midline incision that incorporates or excises the prior surgical scar is made extending between the tibial tubercle to the distal portion of the *QT* (typically, this is extended proximally for *QT* retraction). Medial and lateral skin flaps are elevated to visualize the retinaculum and vastus medialis and lateralis. Atrophic tendon ends are debrided to healthy tissue, with large gaps expected (10-15 cm in our cases).

**Figure 3:**
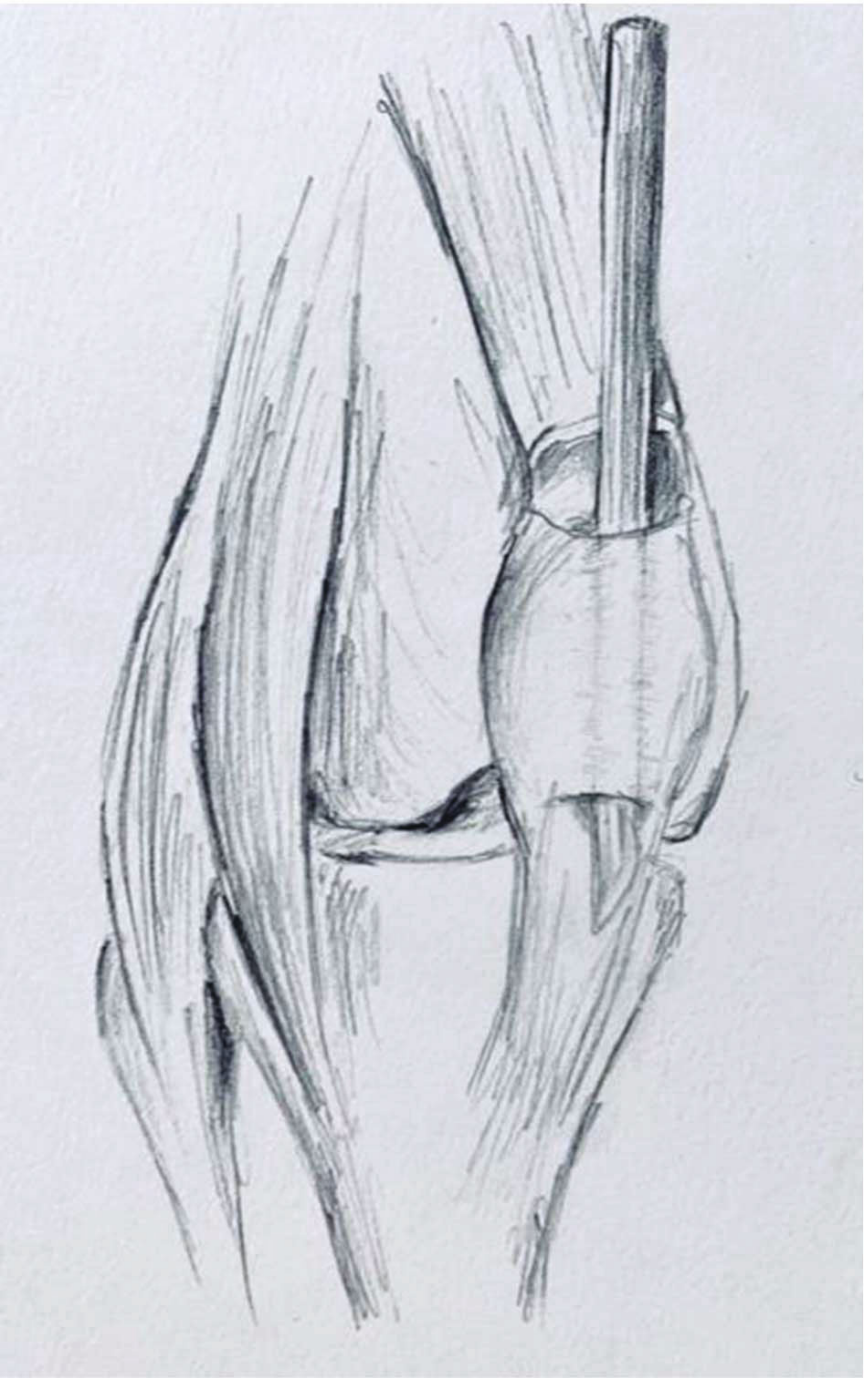
Illustration of preparation of subperiosteal patellar tunnel.

**Figure 4:**
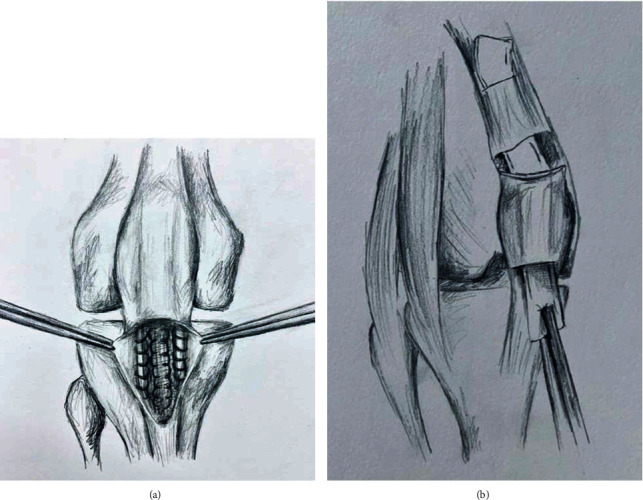
(a) Illustration of mesh fixation distally into patellar tendon and deep to the paratenon layer. (b) Overview of passage of mesh subperiosteal over patella and into quadriceps tendon.

**Figure 5:**
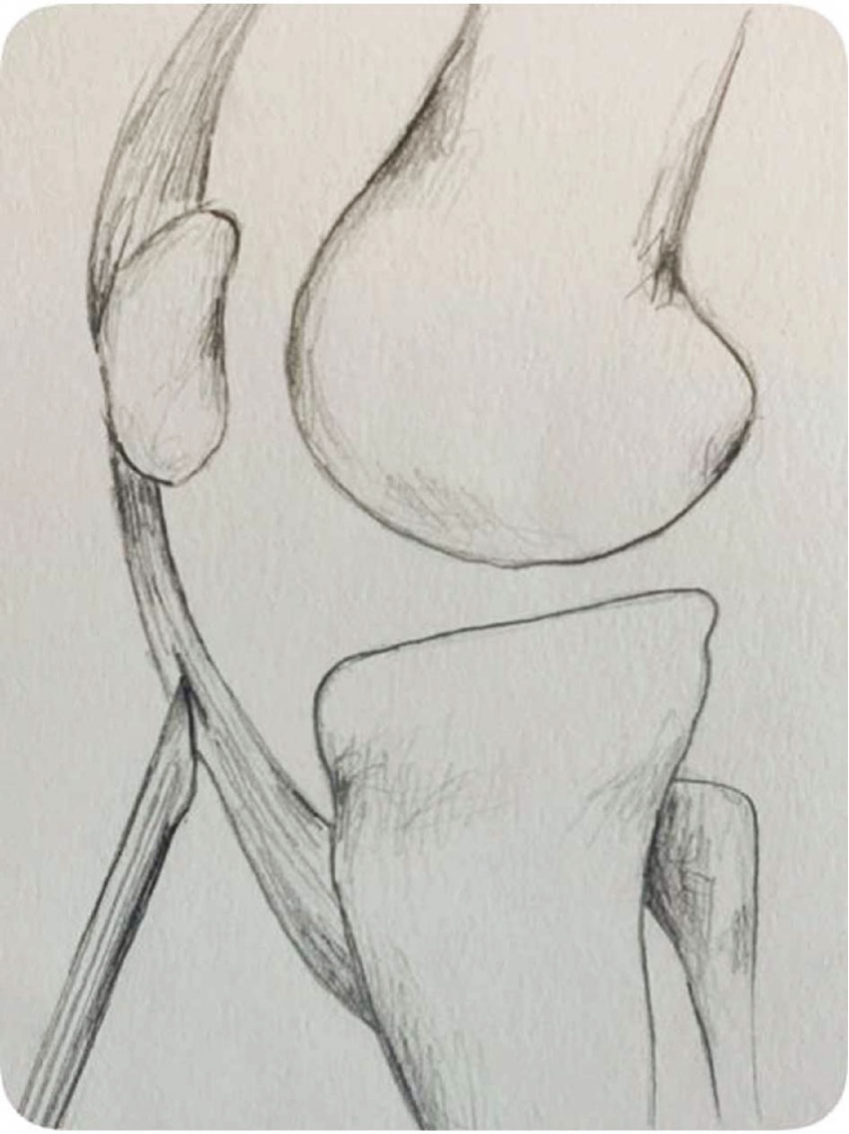
Illustration of preparation of transpatellar tendon tunnel for mesh passage and distal fixation.

**Figure 6:**
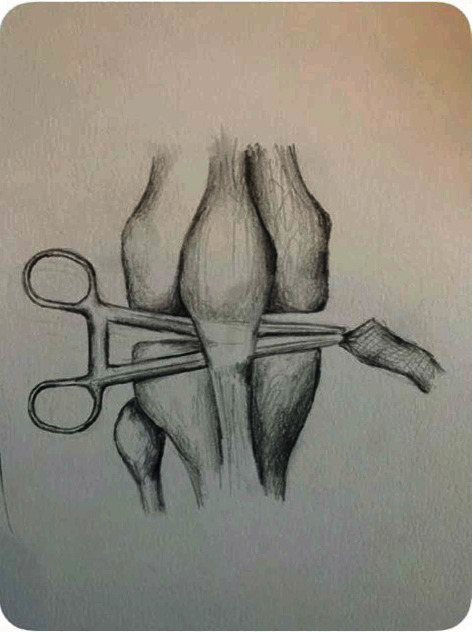
Illustration of passage of mesh through created patellar tunnel.

**Figure 7:**
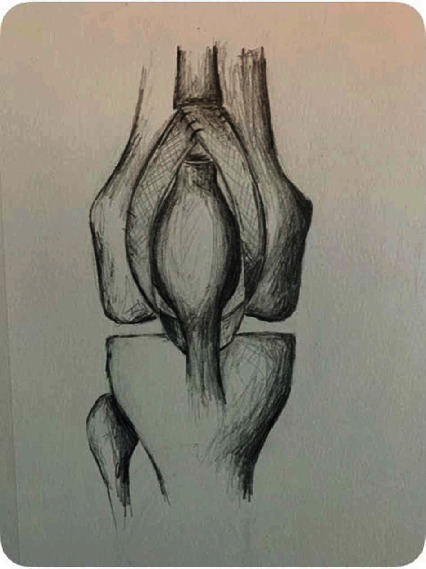
Illustration of overall mesh fixation construct through the patellar tendon distally and around medial and lateral sides of patella with fixation into proximal quadriceps tendon.

The mesh (Covidien macroporous polypropylene mesh, 45 × 30 cm) is tubularized as previously described [10], measuring 2 cm × 30 cm. We employed two distal fixation techniques. In case 1, a subperiosteal tunnel was created over the anterior surface of the patella (Figure 3). Distally, the paratenon overlying the patellar tendon (PT) was incised and reflected. The mesh is then passed subperiosteally over the anterior patella and incorporated onto the PT with Krakow suture fixation (Figures 4(a) and 4(b)). The paratenon layer is then repaired over the mesh, similar to prior reports [11]. In case 2, the mesh captures the patella distally using a transverse tunnel through the PT, 1 cm distal to the inferior pole of the patella (Figure 5). The mesh is passed through the tunnel (Figure 6) in a loop fashion and sutured to each side of the quadriceps tendon proximal to the patella, using a Krakow suture technique (Figure 7). These techniques differ from previously described techniques in knee arthroplasty that relied on intraosseous graft fixation in the tibia for distal fixation [10].

**Figure 8:**
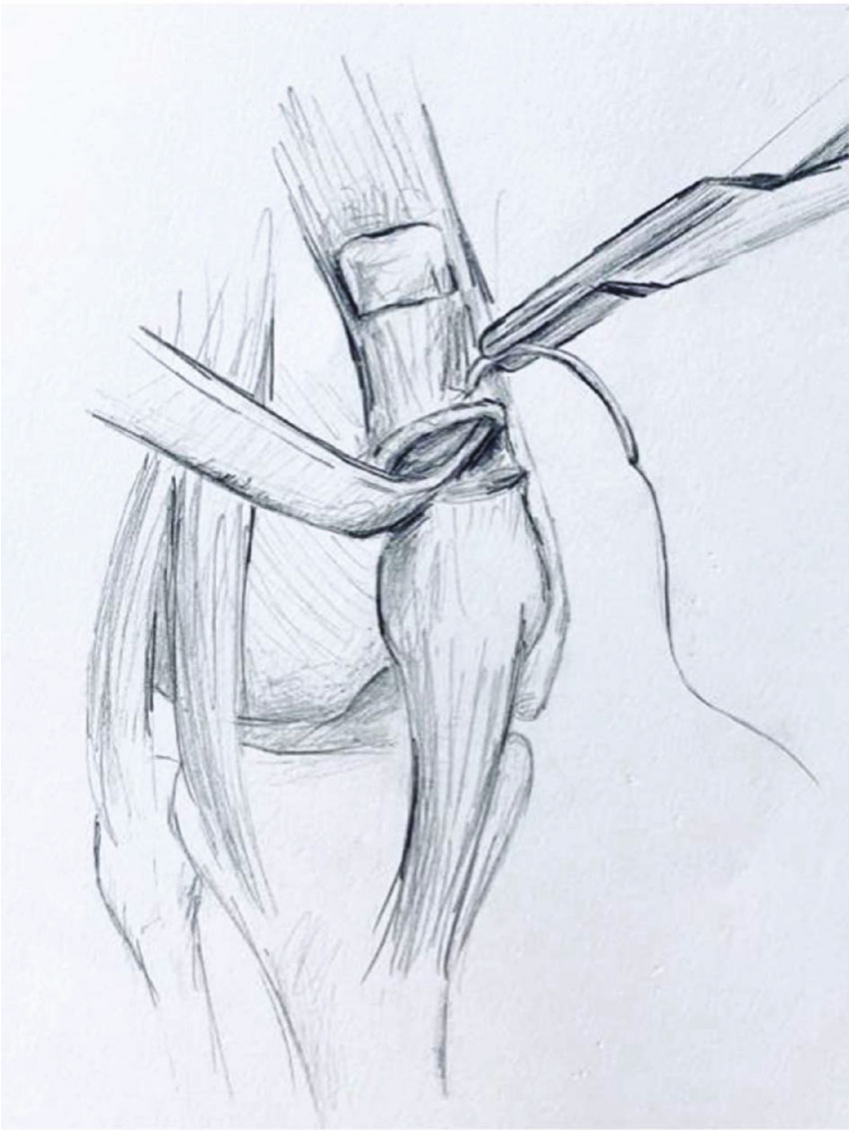
Illustration of proximal mesh fixation and preparation of remaining quadriceps tendon.

Proximally, an intrasubstance, longitudinal tunnel is made in the remnant quadriceps tendon stump, and the mesh is secured (after reapposition and tensioning) to the tendon with running, Krakow suture technique (Figure 8). For all cases, the *QT*-mesh unit is tensioned tightly with the knee in full extension.

Deep wound closure should ensure complete coverage of the synthetic mesh when possible. Proximally, this includes mobilization of the vastus medialis and lateralis myofascial units for coverage, as previously described [10]. Distally, the closure includes closure of the paratenon (case 1) or retinaculum (case 2).

#### 2.1.4. Postoperative Protocol

Postoperatively, patients are weightbearing as tolerated in a removable hinged knee brace locked in extension for three months, after which flexion limits are increased (via the brace) in 30° per 2-week intervals. Upon achieving 90° of flexion, ROM is progressed to tolerance. It is important to avoid active or passive knee flexion for a prolonged time.

## 3. Discussion

*QT* ruptures exceed patella fractures and PT ruptures in their incident disruption of the knee extensor mechanism [12]. When not treated acutely or after failure of primary repair, *QT* tears become increasingly difficult to treat. In this series, we highlight two successful reconstructions in *QT* deficient native knees utilizing a synthetic mesh augmentation for reconstruction. While many methods have been described to reconstruct irreparable *QT*s, our small series demonstrates a reliable alternative to soft tissue reconstructions. *Classically, techniques such as the Codivilla or Scuderi advancement techniques are effective for reconstruction of smaller QT gaps than we encountered (10-15 cm)* [13]. Despite the array of autograft, allograft, and synthetic surgical augmentations, outcomes remain suboptimal [14–19]. Unlike customizable synthetic grafts, auto- and allografts have unique risks that include graft-host mismatch [20], reliance on graft tissue quality, donor site morbidity (in the case of autograft), risk for delayed creep failure, allograft availability, and disease transmission. *However, when possible, utilization of autograft represents the most cost-effective source for extensor mechanism reconstruction tissue*.

Given concerns over the risk-success profile of soft tissue graft reconstruction, we adapted a TKA reconstruction technique [10], applying a synthetic mesh augmentation for the reconstruction of chronic extensor mechanism disruptions with good outcomes [21–24]. This augmentation technique was previously modified to augment acute, native *QT* repairs with good success [25]. It has also been described to augment an allograft chronic *QT* reconstruction [13] but has never been portrayed in isolation. Monofilament mesh is well-studied in general surgical hernia repairs 26 and functions by inciting a robust inflammatory fibrotic reaction that promotes host/graft integration [27, 28]. A TKA retrieval study demonstrated similar histological findings [29]. The use of mesh in this technique is technically uncomplicated and affordable, and polypropylene mesh has favorable biomechanical properties [13, 29]. As such, there is growing interest in its use in the traditionally tenuous reconstruction of relatively devitalized post-TKA extensor mechanism ruptures [10, 21, 30, 31].

We selected this method for these two patients given their unique circumstances: chronicity, kidney disease in a high-functioning patient (case 1), and high-demand knee reliance in a contralateral amputee (case 2). Thus, both patients demanded a reliable method for recalcitrant chronic *QT* ruptures. As such, reconstruction in this setting likely represents an approximate worst-case scenario [22], and success would seem relatively promising for adaptation to similarly exacting pathology [24, 32].

The success of this technique relies heavily on distal mesh fixation with described techniques including screw and cement fixation in the tibial plateau [10, 21, 24] and suture fixation into the PT [11, 23]. While most prior literature describes fixation in postarthroplasty knees, our technique demonstrates practicality in the native knee with chronic *QT* disruption. While the proximal fixation has limited flexibility, we demonstrate two successful distal fixation methods, including a self-retaining sling and direct tendon onlay through a subperiosteal tunnel. We believe that avoiding any knee flexion for a prolonged time period after surgery is critically important. This can be achieved with a cylinder cast [22] or with a knee immobilizer in a reliable patient.

## 4. Conclusion

Chronic *QT* tears that have failed primary repair have notoriously poor results. Multiple options exist, but synthetic mesh has emerged as an option for reconstruction in the arthroplasty patient. This report demonstrates its viability in the native knee and offers a technical description of two distal fixation methods. Longitudinal investigations should quantify and compare the efficacy of this novel technique; however, we hope it provides a usable alternative to graft reconstruction for chronic tendon injuries given our success here and in prior descriptions after TKA.
